# The coordination of nuclear envelope assembly and chromosome segregation in metazoans

**DOI:** 10.1080/19491034.2020.1742064

**Published:** 2020-03-25

**Authors:** Shiwei Liu, David Pellman

**Affiliations:** a Howard Hughes Medical Institute, Chevy Chase, MD, USA; b Department of Cell Biology, Harvard Medical School, Boston, MA, USA; c Department of Pediatric Oncology, Dana-Farber Cancer Institute, Boston, MA, USA

**Keywords:** nuclear envelope, nuclear pore complex, mitosis, chromosome segregation, micronucleus

## Abstract

The nuclear envelope (NE) is composed of two lipid bilayer membranes that enclose the eukaryotic genome. In interphase, the NE is perforated by thousands of nuclear pore complexes (NPCs), which allow transport in and out of the nucleus. During mitosis in metazoans, the NE is broken down and then reassembled in a manner that enables proper chromosome segregation and the formation of a single nucleus in each daughter cell. Defects in coordinating NE reformation and chromosome segregation can cause aberrant nuclear architecture. This includes the formation of micronuclei, which can trigger a catastrophic mutational process commonly observed in cancers called chromothripsis. Here, we discuss the current understanding of the coordination of NE reformation with chromosome segregation during mitotic exit in metazoans. We review differing models in the field and highlight recent work suggesting that normal NE reformation and chromosome segregation are physically linked through the timing of mitotic spindle disassembly.

## Introduction

**Figure 1.**
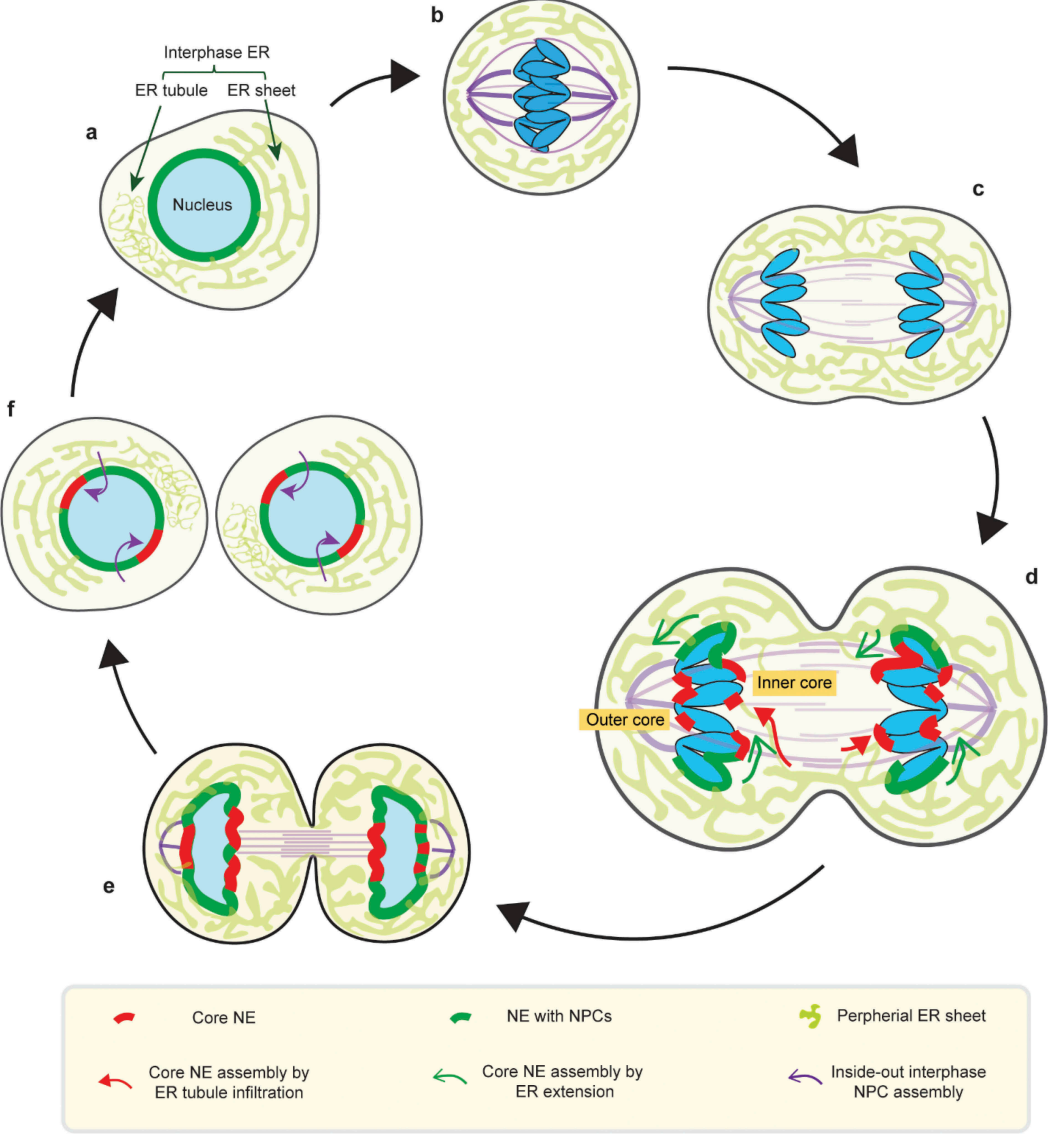
Dynamics of the NE during the normal cell-cycle. (a) Interphase NE and ER organization. The interphase ER is continuous the NE and forms an interconnected network of membrane sheets and tubules. (b) In metaphase, the NE is absorbed into the mitotic ER, which is largely excluded from the spindle (purple). (c) Similarly, the mitotic ER remains largely shielded from the anaphase spindle during chromosome segregation. (d-e) (also see section 3 for details) In telophase, segregated chromosome masses recruit membranes to reform the NE. The chromosome regions in contact with the spindle assemble the core NE (thick red lines), whereas the chromosome peripheral regions assemble the non-core NE with NPCs (thick dark green lines). The core membranes abutting the central spindle are termed the ‘inner core’; the core membranes abutting the spindle pole and its microtubules are termed the ‘outer core’. (d) Two hypothetical models for the delivery of (core) membranes into the anaphase/telophase spindle (see section 5 for details): 1. core membrane delivery by direct ER tubule infiltration (red arrows); 2. core membrane delivery by extension of the nascent NE from chromosome periphery/non-core domain (green arrows). (f) (see section 3) In the subsequent interphase, the core NE initially lacking NPCs forms pore-free islands, which progressively assemble NPCs through an inside-out mechanism (purple arrows).

During mitosis in metazoans, the nuclear envelope (NE) (Figure 1(a)) partially or completely breaks down to permit the assembly of a spindle in the mitotic cytoplasm (Figure 1(b)). NE breakdown (NEBD) is primarily promoted by the activation of mitotic kinases at mitotic entry, phosphorylating many NE proteins, including various inner nuclear membrane (INM) integral proteins, nuclear lamins, and nuclear pore complex (NPC) subunits (also termed nucleoporins or NUPs) [1,2]. Mitotic phosphorylation of NE proteins disrupts protein complexes and dissolves connections between the nuclear membrane and mitotic chromosomes. The clearance of membrane from chromosomes, along with the disassembly of NPCs into subcomplexes, temporarily disrupts nuclear compartmentalization [1,2]. Accompanying these events, the nuclear membrane is absorbed into the endoplasmic reticulum (ER) [3,4], which is largely excluded from the spindle region [5–8] (Figure 1(b)). This ER exclusion may involve an active clearance of membranes mediated by the motor protein dynein, the LINC (linker of nucleoskeleton and cytoskeleton) complex embedded in the NE, and microtubule-binding ER proteins, REEP3/4 (receptor expression-enhancing protein 3/4) [9–11]. Alternatively, or additionally, passive ER exclusion by spindle microtubules is thought to occur when mitotic phosphorylation of ER-microtubule linking proteins disrupts their interaction with the spindle [7,12]. While the importance of ER membrane exclusion from the spindle is not clearly established, it has been proposed to facilitate movement of mitotic chromosomes [11,13], including metaphase chromosome congression and anaphase segregation of chromosomes [14] (Figure 1(b-c)).

In telophase, the two segregated masses of chromosomes recruit membranes and NE proteins, including NPCs, to reform interphase nuclei (Figure 1(d,e)). Recruitment is enabled by reversing the mitotic phosphorylation of NE proteins by phosphatases [1,2]. However, nuclear envelope reformation (also referred to as NE reassembly) is not simply the reversal of NEBD. Unlike NEBD, where both the nuclear membrane and NPCs can, in principle, directly disassemble from chromosomes [1,2,15], NE reassembly occurs when telophase microtubule bundles impinge on the chromosome masses [2,5,16,17] and can interfere with membrane access to chromosomes (Figure 1(d)). Because each segregated chromosome is transported by the spindle independently [14,18], enclosure of all the chromosomes in a single nucleus requires that the recruitment of the newly forming nuclear membrane must, in some way, be coordinated with spindle disassembly.

Defects in coordinating NE reassembly with spindle disassembly can lead to impaired nuclear architecture and impaired nuclear function. For example, mitotic errors that generate dicentric or lagging chromatin result in the formation of abnormal nuclear structures, chromosome bridges or micronuclei [19,20]. These structures often exhibit DNA replication defects and nuclear envelope fragility, ultimately leading to genome instability [21–29]. Moreover, the NE of these structures is typically depleted for many important proteins, including B-type lamins and NPCs [21–23,25], suggesting that their impaired nuclear function could result from altered NE protein composition.

The paucity of NPCs on these abnormal nuclear structures appears to be caused by defective NPC assembly during nucleus reformation [23,30]. At the end of mitosis (telophase) prior to complete enclosure of chromosomes by membranes, thousands of NPCs are incorporated into the assembling nuclear membrane with remarkable rapidity and synchrony (a < 10 min interval) [31,32]. Failure to assemble NPCs during this time (e.g. by depletion of key NPC proteins for NPC assembly in *Xenopus* egg extracts), results in formation of a double nuclear membrane around chromosomes [33–37] that is irreversibly defective in acquiring NPCs [33]. Prior studies suggested that the next wave of NPC assembly during interphase requires preexisting NPCs derived from postmitotic NPC assembly (see ‘interphase NPC assembly mechanism’ in the section 3 below for details) [32]. As such, abnormal nuclear structures formed with reduced NPCs, such as micronuclei or chromatin bridges, exhibit lower numbers of NPCs throughout interphase [21,23,25], a defect which impairs nuclear processes such as transcription and DNA replication. Therefore, getting the initial burst of telophase NPC assembly ‘right’ is essential for normal interphase nuclear function.

In this review article, we review proposed mechanisms for the coordination of NE and NPC assembly with chromosome segregation. First, we discuss mechanisms that may promote the formation of a single nucleus with a normal complement of NPCs (section 2). Next, we discuss circumstances where the assembly of the nuclear membrane and the assembly of NPCs can be uncoupled, both during normal chromosome segregation (section 3) and in the context of chromosome mis-segregation, generating micronuclei (section 4). We then discuss contrasting models to explain this uncoupling (section 5). Finally, we discuss mechanisms that may impact genome integrity by influencing the rate of micronucleation (section 6). We highlight our recently proposed model that the coordination between NE assembly and chromosome segregation results simply from the physical organization of mitotic cells: the presence and timely disassembly of the mitotic spindle and the organization of the mitotic ER network [23].

## Coordinating membrane and NPC assembly during mitotic exit

Interesting exceptions aside [38–40], eukaryotic cells generally contain only one nucleus. Although it is not precisely clear why this is the case, there are a number of possibilities. First, a single nucleus is necessary for any DNA repair reaction that uses the homologous chromosome as a template [41], as no recombination can occur between chromosomes encapsulated within separated nuclei. For example in meiosis homologous chromosomes are brought together in the prophase I nucleus, synapsed and then undergo programed inter-homologue recombination [42]. Second, having a single nucleus may also facilitate transcriptional regulation by non-coding RNAs acting in trans [43]. Third, a single nucleus may be more economical in terms of lipids and NE proteins [44], due to the reduced surface area of a single nucleus compared to multiple nuclei of the same volume. Fourth, if cells contain limiting amounts of nuclear proteins (e.g. transcription factors or NE proteins), having a single nucleus should minimize competition between nuclei for these components. In principle, this could dampen noise and reduce asynchrony in nuclear processes such as transcription and DNA replication [45]. Finally, a single nucleus may promote the proper balance in assembling non-membranous nuclear sub-organelles that are important for many nuclear activities. Therefore, understanding how a single nucleus forms has broad implications, touching on many aspects of cellular function.

Several mechanisms are known to promote the formation of a single nucleus. First, the ensemble segregation of anaphase chromosomes is facilitated by a complex signaling mechanism called the spindle assembly checkpoint, which ensures correct microtubule attachments to mitotic chromosomes [46]. In part through the concerted action of motor proteins [14,47,48], correctly attached chromosomes are kept in close physical proximity as they move toward the spindle poles during anaphase. This clustering of chromosomes is an important physical characteristic of mitosis that promotes the formation of a single nucleus. A second important factor is the temporal control of NE reassembly. Due to the timing of the inactivation of mitotic cyclin-dependent kinase activity (CDK1) [49–52], NE reassembly normally occurs in telophase, after anaphase chromosome segregation is complete. Therefore, straggler, late-segregating chromosomes that ‘lag’ behind the other chromosomes during early or mid-anaphase still have the opportunity to catch up to the other chromosomes prior to the start of NE assembly. Lastly, recent work has demonstrated that when membranes enclose chromosomes, a DNA crosslinking protein called barrier-to-autointegration factor (BAF) restricts these membranes to the surface of the chromosome mass, preventing nuclear membrane infiltration into the space between individual chromosomes [18]. Therefore, the packaging of chromosomes into a single nucleus does not simply occur by passively re-tethering membranes to chromosomes, but rather is mediated by a series of regulated steps.

The assembly of a functional nucleus requires the incorporation of NPCs. Upon mitotic entry, NPCs are disassembled and dissociated into mostly soluble subcomplexes from membranes. During mitotic exit, NPCs, which are highly complex in structure, are rapidly regenerated on the nascent nuclear membrane [1,32]. The rapidity of the process poses experimental challenges for its study, which has led to much debate about the underlying mechanism. It is clear that the concentration of nascent NPCs on chromatin is mediated by the chromatin binding NPC component, ELYS (embryonic large molecule derived from yolk sac) [34,36,37,53,54], in concert with the high local concentration of activated RanGTPase [55–58]. Therefore, an early proposal was that NPC assembly starts with the oligomerization of an ELYS-containing NPC subcomplex (the NUP107-160 subcomplex) on decondensing chromosomes in anaphase, followed by the recruitment of membranes in telophase [59]. However, later studies have suggested that the ELYS/NUP107-160 subcomplex does not oligomerize on chromatin in the absence of membranes [5,55], suggesting that the linkage of nucleoporins to chromatin and membranes might occur near-simultaneously, perhaps in a concerted manner. In accordance with this view, a recent time-resolved electron microscopy (EM) analysis of human HeLa cells shows that NPCs are formed through radial dilation of nucleoporins residing within individual small membrane fenestrations of the peripheral ER sheets, when these ER sheets enclose chromosomes in telophase [31] (Figure 1(c,d)). Interestingly, this study also shows that in anaphase, membrane fenestrations are already abundant on peripheral ER sheets prior to their recruitment to chromosomes [31]. Thus, prefabricated fenestrations within membrane sheets that readily incorporate nucleoporins may explain the rapid and synchronous NPC assembly during mitotic exit, as it may dispense with the need for *de novo* nuclear membrane fusion to produce membrane pores.

How do nucleoporins know when and where to assemble? One mechanism may involve the curvature-sensing ability of nucleoporins (e.g. NUP133 within the NUP107-160 subcomplex [37]), which might facilitate their targeting to membrane fenestrations [32]. Additionally, signals from mitotic chromosomes guide the deposition of nucleoporins to the reforming nuclear membrane. These signals include the high RanGTP level around mitotic chromosomes as well as the binding of ELYS to chromatin [34–37,53,55,56,58]. Supporting this idea, disruption of the RanGTP gradient or depletion of ELYS directs NPC assembly to the ER, forming annulate lamellae containing ectopic cytoplasmic pore complexes [37,56,60]. Interestingly, during early *Drosophila melanogaster* embryogenesis, annulate lamellae are developmentally programmed to form and are used as a reservoir of incompletely assembled NPCs to supply NPC precursors for NE formation during mitotic exit [61]. This is thought to enable rapid NE reformation during short cell-cycles at this stage of embryogenesis [61]. These incomplete NPCs of annulate lamellae contain ELYS [61]. Therefore, at least in this context, ELYS can also be incorporated into peripheral ER membranes without any contact with chromatin. Whether this association of ELYS with annulate lamellae is then followed by ELYS chromatin-binding to trigger subsequent full NPC assembly remains to be determined. Nevertheless, it is appealing to speculate this process may share some common features with the postmitotic NPC assembly mechanism in normal somatic cells [62]. In summary, the recruitment of the nuclear membrane and NPCs to chromatin are tightly coordinated during mitotic exit to enable the rapid formation of a functional nucleus.

## Transient NE subdomain formation during normal NE reassembly

Although there are mechanisms to promote the concomitant recruitment of the nuclear membrane and NPCs, these processes can be transiently uncoupled in the region of the chromosomes adjacent to the spindle. Whereas the chromosome regions that are not in contact with the spindle are enclosed by NPC-containing NE (the ‘non-core’ NE domain), the NE of microtubule-proximal chromosome regions (the ‘core’ NE domains) is typically strongly depleted of NPCs (Figure 1(d,e)) [16,63–65]. NPC recruitment to the non-core domain assembles approximately half of the NPCs that will eventually be incorporated into the resulting interphase nucleus [31,65]. By contrast, the core chromosome regions initially acquire nuclear membranes and a subset of NE proteins, but not NPCs [65]. Consequently, after mitosis, the NE is inhomogeneous: by current thinking, the former core NE domain becomes interphase ‘pore-free islands’ whereas the former non-core domain contributes the remainder of the NE with NPCs (Figure 1(f)) [65,66].

As interphase progresses, these pore-free islands are slowly populated with NPCs [65–68]. Unlike fungi, NPCs in metazoan nuclei have limited mobility partly due to interactions with the nuclear lamina [69,70]. Accordingly, pore-free islands acquire NPCs via a second, *de novo* NPC assembly mechanism, which approximately doubles the number of NPCs. The NPC assembly during interphase occurs with much slower kinetics compared to postmitotic NPC assembly and has different genetic requirements [37,71–77]. Several factors are thought to contribute to the slow kinetics of interphase NPC assembly. One contribution may come from the requirement for *de novo* nuclear membrane fusion during NPC insertion into the intact interphase nuclear membrane [32]. In human cells, membrane deformation has been observed to initiate from the nucleoplasmic side of the inner nuclear membrane (INM) and proceeds through an inside-out extrusion mechanism (Figure 1(f)) that culminates in fusion of the inner and outer nuclear membranes (INM and ONM, respectively) [65]. This inside-out assembly mechanism presumably requires that NPC components are first imported into the nucleus [71], meaning that the process of filling in the pore-free islands relies on some functional NPCs in adjacent regions of the nucleus [76] (Figure 1(f)). Consistent with the idea that preexisting NPCs are required for interphase NPC assembly, prior *in vitro* data demonstrates that if chromosomes are enclosed in an NE lacking NPCs, the NE becomes irreversibly defective: no new NPCs can be inserted into an NE lacking preexisting NPCs [33]. Another factor contributing to the slow speed of interphase NPC assembly is the asynchrony of the process [65,77].

Interestingly, the paucity of NPCs in the core domains is accompanied by a distinct pattern of abundance of other NE proteins (Figure 1(d,e). Specifically, the core domains become enriched for the group of ‘core NE proteins’, which include BAF, LAP2α (lamina-associated peptide 2α), emerin and a pool of lamin A/C [16,18,63,64,78,79]. By contrast, the non-core domain on the chromosome periphery, contains NPCs and other ‘non-core NE proteins’, such as LBR (lamin B receptor) and lamin B [78,79]. The transient partitioning of the NE in telophase into core and non-core domains is eventually converted into a homogeneous NE later in interphase, in part by NPC assembly in pore-free islands and perhaps also by some intermingling of these membrane subdomains [65–67]. We note that, for unclear reasons, the distinct separation of core and non-core NE domains is more obvious in some cell lines/experimental conditions than others [23]. Perhaps due to differing extents in separation of the core and non-core NE domains, the morphology of the resulting pore-free islands has also been reported to differ between cell types [68].

Although non-core proteins are generally excluded from the core domain, we emphasize that the non-core domain actually contains both core and non-core proteins, although the abundance of the core proteins in the non-core domain is lower than in the core. This is most evident when the nascent nuclear membrane initially encloses chromosomes. During this initial stage, core proteins accumulate around the entire chromosome mass [16,18,63,64]. Shortly thereafter, most core proteins (e.g. BAF, LAP2α and emerin) become concentrated to higher levels in the core domain [16,18,63,64].

What establishes the transient partitioning of the NE into core and non-core domains? One possibility is that the core NE and non-core NE might originate from different segments of the membrane network that have different morphologies. Although the membrane-associated core and non-core proteins are thought to largely co-localize throughout the continuous mitotic ER before NE assembly [3,4,6], there may be differences in the way that differing ER morphological structures (sheets versus tubules [5,6,31,80–82]) interact with chromatin during NE assembly. For example, NPCs may only be able to assemble on fenestrated sheets and, as discussed in **section 5** below, the spindle may inhibit the access of these sheets to the core chromatin region. By contrast, core proteins may be able to access both ER sheets and small, thin ER tubules. These differences may be amplified by the fact that the localization of some NE proteins is dependent upon other NE proteins. For example, the exclusion of LBR from the core domain may be due to the local depletion of nucleoporin ELYS, which was proposed to promote the recruitment of LBR [83,84]. Similarly, the enrichment of core proteins in the core domain might be due to the absence of NPCs, which import the vaccinia-related kinase 1 (VRK1) that inhibits BAF assembly on chromatin [85–87]. Diminished VRK1 should stabilize DNA-bound BAF, increases its local abundance, leading to the recruitment of other BAF-interacting, LEM domain-containing core proteins. Although this general model has some experimental support, much remains unknown about the molecular mechanism underlying the formation of NE subdomains.

The functional significance of the transient NE subdomain formation is also not entirely clear. Early studies had proposed that the core protein BAF may selectively direct the recruitment of core protein-containing membranes to chromosome regions, circumventing microtubule-interference with the recruitment of non-core protein-containing membranes [16]. Although it has been recently shown that BAF is not required for membrane recruitment [18], as proposed in the original model [16], the concentration of core proteins near microtubules may nevertheless assist spindle disassembly and facilitate NE enclosure. First, emerging evidence indicates that sealing of the core nuclear membrane is coupled with local microtubule severing by spastin and perhaps other proteins associated with the endosomal sorting complexes required for transport-III (ESCRT-III) [17,88]. Second, the recruitment of ESCRT-III to the core domain requires a LEM (LAP2-emerin-MAN1) domain-containing protein called LEM2 [89,90], which undergoes a phase separation-mediated enrichment in the core domain in a microtubule-dependent manner [91]. As BAF is immobile when enriched in the core domain [16,92], it is possible that BAF also promotes the LEM2 enrichment. Supporting this idea, the LEM domain of LEM2, which interacts with BAF, is required for the LEM2 concentration at the core [91]. In summary, the core domain may not just passively form as a consequence of NPCs and other non-core proteins being unable to assemble on membranes near the spindle; instead, the core domain may, “by design”, be formed to assemble NE on chromatin abutting the spindle and have specific functions in promoting local NE integrity and spindle microtubule disassembly.

## The pattern of NE and NPC assembly when chromosomes are mis-segregated

The mechanisms described above facilitate normal nucleus formation. Nevertheless, mitotic errors can cause a variety of nuclear abnormalities. For example, merotelic kinetochore attachments, where a single kinetochore is bound by microtubules originating from both spindle poles, are not well sensed by the spindle assembly checkpoint [46]. If uncorrected, merotelic-attachments cause chromosomes to lag near the spindle equator, with some ‘laggards’ persisting until NE assembly occurs in telophase [23,93–95]. The physical separation of lagging chromosomes from the main mass of chromosomes causes the formation of micronuclei. A variety of other mitotic errors can also produce micronuclei, as can DNA breaks that generate acentric chromosome fragments [20]. Micronuclei are common in many contexts including cancer [20,96]. Recently, studies from us and others indicate that micronuclei are sources for a catastrophic mutational process, chromothripsis, where massive chromosome rearrangement occurs on only one or a few chromosomes [19,24,26,27,97]. Chromosomes in micronuclei become damaged, at least in part, after the sudden loss of nuclear envelope integrity [21–24,27]. Because of this relevance to genome stability, the basis for the NE fragility of micronuclei has been an important problem.

Although NE/NPC assembly on the normally segregated chromosome mass is actively studied, surprisingly little was known about the spatiotemporal pattern of NE/NPC assembly on mis-segregated chromosomes until recently. Several studies have now established that mis-segregated chromosomes undergo altered NE/NPC assembly, although the details of the observations and the interpretation of the results significantly differ between these studies. Below, we briefly summarize these studies.

**Figure 2.**
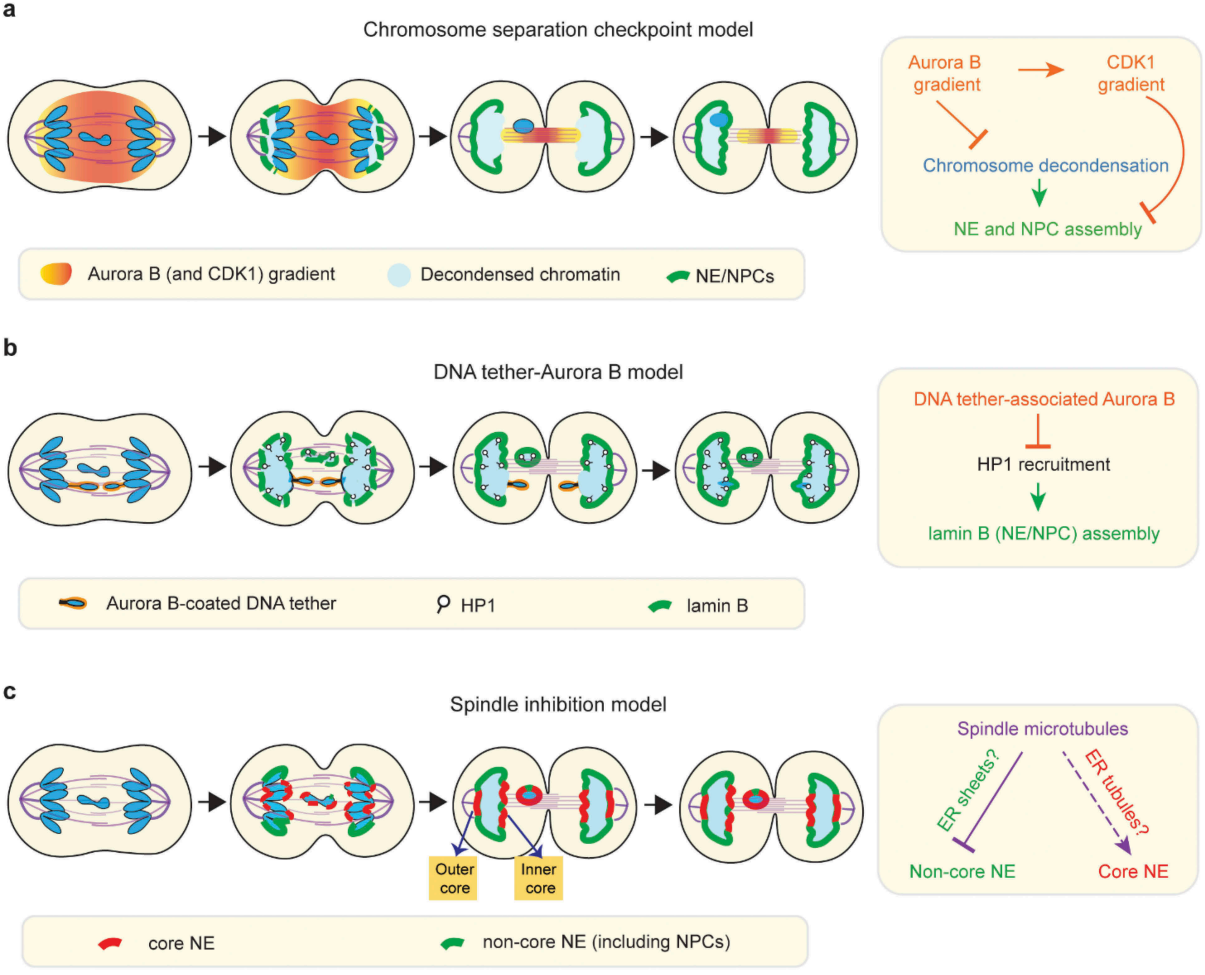
Models for NE/NPC assembly on mis-segregated chromosomes. (a) The chromosome separation checkpoint model: NE and NPC assembly is locally delayed on chromosome regions in proximity to the midzone phosphorylation gradient of Aurora B and CDK1 (see section 5 for details). Under this model, Aurora B activity inhibits NE/NPC assembly by enforcing chromosome condensation. In addition, NE/NPC assembly is inhibited by a gradient of CDK1 activity that mirrors the Aurora B gradient. The chromosome separation checkpoint model was proposed to correct mis-segregated chromosomes. Under this model, mis-segregated lagging chromosomes can be reintegrated through the inner core membrane gaps of the reforming primary nucleus. Accordingly, as the outer core is located furthest to the midzone Aurora B, it is expected to be the first region to assemble NE/NPC as illustrated in the cartoon here. However, at least in some human cell lines, the outer core is often depleted for NPCs during NE assembly (see c). (b) The ‘DNA tether-Aurora B model’: NE/NPC assembly is not delayed on the intact lagging chromosome, but it is specifically delayed on acentric fragments that are connected to the main chromosome mass by DNA tethers. Under this model, the pool of Aurora B coating on DNA tethers inhibits NE/NPC assembly by blocking HP1 recruitment (see section 5 for details). (c) The ‘spindle inhibition model’: on lagging chromosomes, the non-core NE (with NPCs) assembly is inhibited by the mitotic spindle whereas the core NE assembly is either not affected or less affected.

Using nucleoporins (e.g. NUP107) and lamin B to track postmitotic NE assembly, Afonso *et al*. reported that in *Drosophila* S2 cells, lagging chromosomes undergo a pronounced delay in NE and NPC assembly relative to the main chromosome mass [49] (Figure 2(a)). The study concluded that this delay is enforced by a ‘chromosome separation checkpoint’, which uses the midzone phosphorylation gradient from the mitotic kinase Aurora B to monitor chromosome position and prevent NE assembly on incompletely separated chromosomes [49,98] (Figure 2(a)). Accordingly, chromatin regions on the main chromosome mass that face midzone Aurora B (the so-called ‘inner core’ (Figure 1 (d)) were also reported to have delayed NE and NPC assembly [49,98]. This was proposed to generate a membrane gap on the reforming main nucleus through which membrane-free lagging chromosomes might slip through to rejoin the main mass of chromosomes [49,98]. While prior work had suggested that anaphase entry, regulated by the spindle assembly checkpoint, was the final chance to correct mitotic errors, this chromosome separation checkpoint was proposed to sense and correct chromosome mis-segregation later, during mitotic exit to prevent micronucleation [49,98]

A separate study from Karg *et al*. using *Drosophila* neuroblasts reached a somewhat different conclusion [99]. Karg *et al*. used targeted nuclease cutting to generate lagging acentric chromosome fragments which remained physically connected to the main chromosome mass by thin DNA threads termed ‘DNA tethers’ [99,100]. Although they observed delayed NE assembly (assessed by lamin B recruitment) on the lagging acentrics and their associated DNA tethers, they reported that whole (kinetochore/centromere-positive) lagging chromosomes did not exhibit any obvious delay [99], in contrast to Alfonso, *et al* [49]. (Figure 2(b)). In common with Alfonso *et al*. and prior work [101], this study implicated Aurora B activity in inhibiting NE assembly on lagging acentrics. However, unlike Alfonso *et al*., this study concluded that Aurora B acts locally from a specific DNA tether-associated pool of Aurora B [99,100] (Figure 2(b)) rather than through an Aurora B gradient extending across the entire anaphase/telophase spindle [49,98,102] (Figure 2(a)). In other words, these two studies differ on whether Aurora B inhibition acts on all chromosomes or only on damaged acentrics and they differ with respect to the location from which Aurora B acts.

More recently, in human HeLa cells, de Castro *et al*. reported that lagging chromosomes can efficiently assemble lamin A/C but cannot recruit NPCs during mitotic exit [30]. Although it was not determined whether these lagging chromosomes exhibited a nuclear membrane assembly delay, their findings are consistent with the possibility that different NE proteins may exhibit different assembly dynamics on lagging chromosomes.

Because the recruitment of nuclear membrane and NPCs (and other NE proteins) can be uncoupled during the normal NE subdomain formation [23,78,79], the differing results from these studies [30,49,99] might be explained, in part, by the use of different protein reporters of NE assembly. Most importantly, none of these prior studies directly assessed nuclear membrane recruitment to lagging chromosomes or the recruitment of core transmembrane proteins such as emerin or LEM2. Therefore, it remained unclear if (and if so, how) the nuclear membrane assembly is altered on mis-segregated chromosomes.

To understand the basis of NE defects of micronuclei, we recently investigated NE assembly around mis-segregated chromosomes in several human cell lines [23]. We analyzed the assembly of both core and non-core NE proteins as well as of the nuclear membrane itself. In contrast to previous work, we found that lagging chromosomes recruit nuclear membrane and core proteins as efficiently as the main chromosome mass with near-normal timing [23] (Figure 2(c)). However, lagging chromosomes failed to normally recruit NPCs and other non-core proteins (e.g. LBR) [23] (Figure 2(c)). The paucity of NPCs persisted through mitosis and, with some variability, was apparent in interphase on the majority of micronuclei generated from these laggards [23]. The NE around lagging chromosomes is therefore much like the core domain, but with the important difference that it is isolated from regions of the NE containing functional NPCs. Like lagging chromosomes, DNA bridges that physically connect telophase chromosome masses also failed to recruit NPCs or LBR properly despite assembling the core membrane [23]. Additionally, other experiments in our study were at odds with the notion of a beneficial Aurora B checkpoint and instead favored the conclusion that NE assembly on lagging chromosomes is irreversibly defective.

## The mechanism(s) for inhibiting non-core NE/NPC assembly on lagging chromosomes

What inhibits the non-core NE/NPC assembly on lagging chromosomes? Or more broadly, what is the mechanism for spatial control of NE/NPC assembly during mitotic exit? Both the chromosome separation checkpoint model [49,98] and the original DNA tether model [99] proposed that NPC and nuclear membrane assembly is inhibited on lagging chromosomes by Aurora B activity. This notion is supported by restoration of NPC (or lamin B) localization to lagging chromosomes after acute Aurora B inhibition around anaphase onset [49,99]. However, the two studies differ in the proposed mechanism for how Aurora B inhibits NE/NPC assembly.

Under the chromosome separation checkpoint model (primarily utilizing *Drosophila* S2 cells), the midzone pool of Aurora B was proposed to block NE/NPC assembly by promoting mitotic chromosome condensation [49,98] (Figure 2(a)). The notion that chromosome condensation might affect NE/NPC assembly was first suggested by work with *Xenopus* egg extracts [101]. In extracts, inhibition of the p97 ATPase prevented p97-mediated extraction of Aurora B from chromosomes, normal chromosome decondensation, and normal NE/NPC assembly [101]. However, whether defective NE assembly was due to defective chromosome condensation or p97 inhibition rather than just being correlated with it was not resolved. In fact, the chain of causality could flow in the opposite direction. Ectopic targeting of a nucleoporin, Sec13, to chromatin can induce local decondensation of polytene chromosomes in *Drosophila* larval salivary glands, suggesting that nucleoporin recruitment or functional nuclear transport may promote chromosome decondensation during mitotic exit [103]. Furthermore, it is not clear that condensed chromatin actually inhibits NPC assembly. Indeed, NE (and NPC) assembly can reportedly occur when chromosome decondensation is delayed. For example, NE assembly has been observed in cell fusion experiments where mitotic cells were fused to interphase cells (a phenomenon called ‘telophasing’) [104] and in cells that undergo prolonged chromosome condensation due to mutations in the Microcephalin (MCPH1) gene [105]. Additionally, using immunofluorescence and EM experiments, we showed that the NE can assemble on condensed chromosomes, including lagging chromosomes [23]. Although it is difficult to exclude a role for subtle differences in the ‘quality’ of chromosome condensation, chromosome condensation *per se* does not appear to be a major barrier for NE/NPC assembly.

More recently, the chromosome separation checkpoint model has been further refined to suggest that a critical Aurora B target, in addition to chromosome condensation, is Cyclin B1/CDK1 [106] (Figure 2(a)). The new study proposes that the midzone pool of Aurora B locally stabilizes Cyclin B1 during anaphase, converting the Aurora B gradient into a midzone-centered CDK1 activity gradient [106]. This midzone pool of CDK1 is postulated to counteract the phosphatase-mediated dephosphorylation of NE proteins such as lamin B, thereby delaying NE assembly on lagging chromosomes [106]. A simple prediction of this model is that both CDK1 and Aurora B inhibition will accelerate NE formation on all segregating chromosomes and restore NE/NPC assembly on lagging chromosomes. However, this is not the case. While small molecule inhibition of CDK1 around anaphase onset, indeed, accelerated NE assembly on the main mass of chromosomes [49,106], it failed to restore NE/NPC assembly on lagging chromosomes [49]. In contrast, Aurora B inactivation around anaphase onset restored NE/NPC assembly on lagging chromosomes but did not accelerate NE assembly on the main mass of chromosomes [49,106]. The opposing effects of CDK1 and Aurora B inactivation suggest that CDK and Aurora B do not act in a concerted manner to regulate NE/NPC assembly.

Under the DNA tether model (from *Drosophila* neuroblast study), the key Aurora B target for inhibiting NE assembly is proposed to be heterochromatin protein 1 (HP1) [107] (Figure 2(b)). Aurora B phosphorylation of histone H3 at serine 10 (pH3S10) prevents HP1 binding to chromatin [108,109]. In principle, this could impact NE assembly because HP1 also binds LBR and could therefore recruit LBR-containing membranes [107,110]. Nevertheless, direct experimental support for HP1-mediated recruitment of LBR and NPC-containing NE is lacking. Moreover, H3 phosphorylation by Aurora B may not generally inhibit NE assembly. In *Drosophila* S2 cells, the expression of an H3 variant that cannot be phosphorylated on serine 10 does not appear to accelerate NE/NPC assembly [49]. Also, in human cells, the Repo-Man/PP1 (protein phosphatase 1) that dephosphorylates pH3S10, is recruited to chromosomes by the nucleoporin NUP153 [111]. This raises the possibility that pH3S10 dephosphorylation and HP1 recruitment could be partly a consequence, not a cause of postmitotic NE/NPC assembly. Irrespective of the generality of the findings on H3 phosphorylation, it is possible that other chromatin modifications and/or the positioning of specific DNA sequences could influence chromatin-NE interactions [112–114] (and E. Hatch, personal communication). Altogether, the potential role of chromatin state in regulating NE/NPC assembly is an interesting topic that remains unclear, but merits further investigation.

An alternative possibility is that Aurora B inhibits NE/NPC assembly indirectly [23], at least in part through its well-known effects on regulating the mitotic spindle [115,116]. In other words, a simple model would be that what inhibits NPC assembly on chromosomes is the spindle itself. Indeed, microtubule stabilization by taxol inhibits NPC-containing NE assembly in human cell lines [5,23] and in *Xenopus* egg extracts [117]. Importantly, this inhibitory effect persists even if Aurora B is inhibited [23,117], meaning that microtubule inhibition of NPC assembly is independent of Aurora B. An inhibitory role for microtubules is also supported by the exclusion of non-core/NPC proteins from the so-called ‘outer core’ of chromosomes in human cells [65,78]. The outer core, as the name implies, is located on the region of the chromosome mass closest to the centrosomes/spindle poles (Figure 1(d)) but furthest from midzone Aurora B (Figure 2(a–c)). Although the formation of an NPC-depleted outer core cannot be explained by the Aurora B gradient, it is simply accounted for by the high density of microtubules near the spindle poles. In Liu *et al*., we acquired evidence that the spindle itself is the primary barrier for recruitment of NPC-containing NE but allows the assembly of membrane containing core proteins [23]. Consistent with much prior literature [16,63,64,83], this explains how the core and non-core subdomains normally form, and also accounts for the striking similarity between the NE on lagging chromosomes and the core domain on normally forming nuclei. For a full discussion of all the evidence supporting a microtubule-inhibition model, we refer the readers to the original study (including our discussion in the bioRxiv preprint) [23,118].

How do spindle microtubules inhibit the non-core/NPC assembly? One possibility is that this may occur through other spindle-associated kinases, such as Polo-like kinase 1 (PLK1) [30]. Given the role of PLK1 in phosphorylating nucleoporins such as NUP98 and NUP53 during NEBD, it is plausible that the spindle-associated pool of PLK1 may locally phosphorylate these nucleoporins and prevent their assembly into NPCs [119]. However, PLK1 is unlikely to be the major regulator because PLK1 inhibition results in only a small restoration of NPCs to lagging chromosomes [30] and this restoration itself might be due to disrupted spindle organization that occurs after PLK1 inhibition [120].

Another possibility is that adjacent to the spindle, there could be a depletion of phosphatases that promote NPC assembly by dephosphorylating nucleoporins. However, relatively little is known about the phosphatases involved and the spatial regulation of their activity. Through an interaction with a kinetochore pool of ELYS [53,121–124], PP1 c, which may dephosphorylate nucleoporins, is recruited to mitotic kinetochores [124]. However, this would concentrate, not deplete, PP1 from the core domain and does not easily explain the lack of NPC assembly within the core domain. Nevertheless, other, as yet unknown mechanisms may promote nucleoporin dephosphorylation during mitotic exit and contribute to the spatial control of NPC assembly.

Overall, we favor a model where the spindle inhibits NPC assembly, at least in part, because of a simple physical barrier effect for the recruitment of ER membranes. During telophase, the chromatin regions that will undergo postmitotic NPC assembly from fenestrated membrane sheets are in proximity to the peripheral ER (Figure 1(d)). By contrast, the chromatin that will be covered by the core NE is initially shielded from the peripheral ER by the spindle [5] (Figure 1(c,d)). We speculate that large fenestrated membrane sheets may therefore have limited access to the chromatin that is adjacent to the spindle. How then does membrane access the core regions? One possibility is that NE-ER membranes may gradually extend from the non-core peripheral region and flow around spindle microtubules (Figure 1(d), Figure 2(c)). Eventually, microtubules are removed by severing enzymes and a continuous membrane would be formed by fusion of smaller membrane structures through the ESCRT-III system [17,88]. Alternatively, or additionally, the membranes populating the core region could directly originate from small thin ER tubules that we speculate might infiltrate the spindle more readily than ER sheets (Figure 1(d), Figure 2(c)). In summary, we propose that NPCs might not assemble in membranes recruited into the typical core region due to a lack the appropriately-sized fenestrations [31] that may be a prerequisite for postmitotic NPC assembly.

## Are there active mechanisms to prevent micronucleation?

The altered spatiotemporal pattern of NE/NPC assembly on mis-segregated chromosomes raises a general question of whether NE assembly and chromosome segregation are actively coordinated. We have discussed mechanisms coordinating these processes and we have also noted that NE assembly typically starts after chromosome segregation is completed, presumably due to the normal timing of CDK1 inactivation. We have highlighted how the timing of the disassembly of the mitotic spindle could impose a loose and error prone mode of coordination between NE assembly and chromosome segregation. However, the possibility of additional checkpoint signaling mechanisms that ‘tighten’ this loose coordination to prevent micronucleus formation could in principle operate. Evidence for such a mechanism, however, is currently sparse.

**Figure 3.**
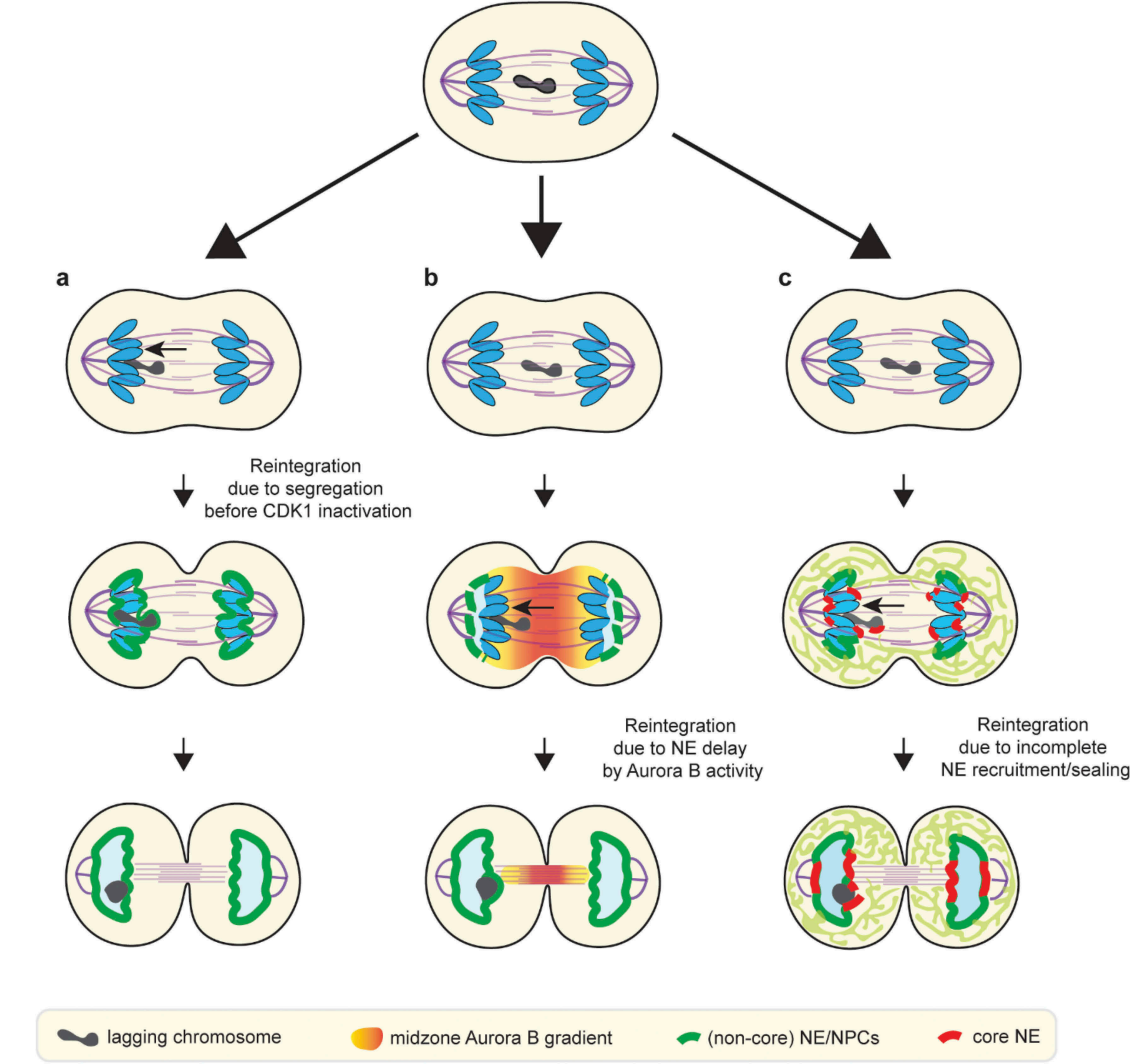
Proposed checkpoint-dependent and checkpoint-independent mechanisms to prevent micronucleation. (a) Lagging chromosome may reintegrate into the main chromosome mass before the main chromosome mass acquires nuclear membrane (checkpoint-independent). (b) Lagging chromosome may reintegrate due to the NE assembly delay induced by the midzone Aurora B activity, as postulated by the chromosome separation checkpoint. (c) Lagging chromosome may integrate due to delayed membrane recruitment/sealing adjacent to the spindle due to the initial ER exclusion from the spindle (checkpoint-independent).

Circumstantial support for a putative checkpoint to prevent micronucleation is based on the observation that many apparently lagging chromosomes reintegrate into the main chromosome mass and do not form micronuclei [125]. However, it is important to distinguish between lagging chromosomes that reintegrate in anaphase, when the chromosome masses have not yet acquired nuclear membrane (Figure 3(a)), from those that reintegrate during telophase, after NE assembly has begun (Figure 3(b,c)). As noted above, NE assembly initiates after anaphase, and is primarily controlled by the timing of CDK1 inactivation [49,52,106]. During anaphase, lagging chromosomes that have made kinetochore-microtubule attachments to opposing poles (merotelic attachments), naturally segregate to one or the other pole due to asymmetry in the number of attached microtubules [93,95]. These chromosomes either join the main mass of chromosomes or wind up close enough to be incorporated together into one nucleus (Figure 3(a)). Therefore, the observation that many lagging chromosomes are incorporated into a single daughter nucleus [47] can simply be explained by timing; it does not, in and of itself, imply that the reintegration is actively promoted by an Aurora B-mediated surveillance/checkpoint mechanism.

Given our current understanding of how NE assembly is regulated, it is also unclear how the cell would actively promote the reintegration of lagging chromosomes into the primary nucleus. The chromosome separation checkpoint model proposes a coordinated delay of NE assembly on lagging chromosomes and on the inner core regions of the reforming primary nucleus to ensure reintegration [49,98] (Figure 3(b)). Like NE assembly on lagging chromosomes, this model proposes that NE assembly at the inner core is inhibited in anaphase due to its proximity to midzone Aurora B [49,98]. However, after anaphase, when the inner core moves away from midzone Aurora B, primary nuclear membrane closure would not be inhibited [49,98]. Once the primary nuclear membrane closes, reintegration of any laggards that persist would be blocked, regardless of the assembly state of the NE on the laggards. Under these circumstances, a checkpoint-mediated correction might still work if there were a dedicated mechanism to ‘re-open’ and fuse the closed nuclear membranes. However, there is at present no clear evidence that this can occur. Indeed, our findings indicated that the NPC assembly defect on laggards becomes largely irreversible after anaphase [23], suggesting the formation of a closed nucleus, consistent with prior work in *Xenopus* egg extracts [33,117]. Moreover, the NE assembly defects persist on micronuclei and we have never observed fusion of micronuclei with the primary nucleus [23] (and our unpublished data).

A recent study from Warecki *et al*. has raised the possibility of an ESCRT-III-mediated membrane fusion event associated the reintegration of nuclease-generated acentric chromosome fragments *Drosophila* neuroblasts [126]. However, in this case, the acentric is connected to the main chromosome mass by a chromatin bridge (‘DNA tether’). As with other chromosome bridges [25,28], membrane closure at the base of the bridge should be blocked by the presence of chromatin. This would leave an open channel to the main nucleus through which the acentric can pass, suggesting that membrane fusion is not necessarily required for reintegration of these tethered acentrics. The specific role of ESCRT and other membrane remodeling proteins in this process merits additional study [126]. Because ESCRT normally seals the spindle-proximal core membranes [17], we speculate that ESCRT on these acentrics might reflect ESCRT's normal function at the core domain: sealing the assembling NE and removing the acentrics-associated microtubule bundles [127], rather than opening up a closed membrane to allow acentric entry.

In summary, we favor the notion that simple physical constraints (e.g. spindle geometry and ER morphology) are the primary link between chromosome segregation and NE assembly. A microtubule-exclusion model can explain the current data on the reintegration of lagging chromosomes and the above described acentrics, without the need to invoke a sophisticated signaling system. For example, in the reforming primary nucleus, the inner core membrane gaps may persist for a longer time interval to permit lagging chromosome reintegration because of timing and geometry rather than checkpoint signaling (Figure 3(c)). First, because the mitotic ER is initially excluded from the spindle [5–8], the rate at which ER membranes access the core region may be influenced by the volume of the spindle, the distance from the core regions to the peripheral ER, and the mechanisms for delivery of ER membranes into the spindle. Furthermore, the inner core membrane gap closure requires membrane sealing by ESCRT-III, the rate of which may depends on the local density of microtubules [17]. Variation in these physical parameters may produce cell-type differences in the timing and the extent of inner core domain formation. Similarly, the position of lagging chromosomes relative to the peripheral ER should also affect the timing of their membrane recruitment. Lagging chromosomes located at the periphery of the spindle, near the peripheral ER, should assemble the NE faster. If these lagging chromosomes are in proximity to fenestrated ER sheets, they may acquire NPCs, as we have recently shown [23]. Therefore, variable position of lagging chromosomes within the mitotic spindle may explain the partial penetrance of the NPC depletion observed on micronuclei [21–23]. Spindle geometry and ER organization vary greatly between cell types [81,128,129], and these variations likely contribute to differences in micronucleation frequencies and the extent of their defects [47,94].

## Implications and future directions

Here, we have reviewed the current thinking about the spatiotemporal coordination of NE/NPC assembly and chromosome segregation. We suggest that the general pattern of NE assembly is established through spatial restriction of the recruitment of NPC/non-core proteins to chromatin by the organization of the mitotic spindle and the mitotic ER network. This restriction may be the consequence of the spindle forming a selective barrier to certain populations of ER membranes. Although this physical model is appealingly simple, more work needs to be done to test the model and to identify the underlying molecular events.

The coordination between chromosome segregation and NE assembly can only be understood if fundamental questions about the mechanism of NE assembly are addressed. For example, there has been spirited debate about whether nascent nuclear membranes originate from ER sheets or ER tubules [5,6,80–82,130]. Inherently, the models are not exclusive, and both populations of membranes could contribute. One simple hypothesis is that postmitotic NPC assembly around the chromosome periphery might be dominated by ER sheets whereas the core NE assembly might have a significant contribution from ER tubules. Apparently conflicting results regarding the contributions of these structures to NE assembly could also be partly explained by cell type-specific differences in the relative abundance of these ER structures [81]. In general, more work needs to be done to understand cell type differences, and in the future, it will be important to study primary cells, preferably in their normal tissue environment.

In addition to a major, poorly understood topic in cell cycle regulation, determining how NE assembly and chromosome segregation are coordinated has important implications for mitotic fidelity and genome integrity. We suggest that the organization of mitotic exit in metazoan cells is not monitored through precise regulatory mechanisms [49,98]. Instead, we favor the view that mitotic exit is inherently error prone, which can explain high rates of micronucleation in tumor cells and rapidly dividing cells of the early embryo [20,94,131]. Due to the absence of strict regulatory controls, mitotic exit may therefore be one of the most vulnerable steps in the metazoan cell cycle, with a high risk for generating abnormal nuclear structures that will then trigger extensive genomic rearrangement [23–25,27,28].
